# Unnecessary Self-defence

**DOI:** 10.1093/ojls/gqaf035

**Published:** 2025-11-12

**Authors:** James Manwaring

**Keywords:** criminal law, defences, self-defence, necessity, necessity requirement, unnecessary force

## Abstract

Self-defence is traditionally said to contain a *necessity requirement*, according to which defensive force is lawful only if it is necessary. But the necessity requirement is formulated inconsistently, and these inconsistencies substantially alter the scope of the defence. This article explains these inconsistencies. It concludes that it would be preferable to abandon the necessity requirement altogether. This would not leave a problematic gap in the law, because necessity would remain an important consideration when judging whether any use of force was reasonable.

## 1. Introduction

It is widely claimed that defensive force is lawful only if it is *necessary*.^1^ Call this the *necessity requirement*.^2^ The necessity requirement can be found at least as early as the 19th century, in which English criminal cases claimed that, to be lawful, force must be used in ‘necessary self-defence’,^3^ or when ‘absolutely necessary’.^4^ The leading 20th-century case held that, when directing juries, ‘All that is needed is a clear exposition … of the conception of necessary self-defence’.^5^ The necessity requirement finds continued support in modern English authorities,^6^ as well as enjoying broad international support, including in the German Criminal Code and US Model Penal Code.^7^ It is also widely defended by moral philosophers and legal scholars.^8^ According to Boaz Sangero:

The requirement that the action of the actor against the aggressor … must be necessary in order to repel the aggressor, is perhaps the most accepted condition of private defence in all the legal systems and from ancient history … Moreover, it is not only that the requirement of necessity is not under dispute, but that it is even widely accepted to see it as a fundamental and significant condition of private defence.^9^

Notwithstanding such claims, the thesis of this article is that the authority supporting a necessity requirement in English law is much weaker than it appears. Further, I will suggest that the law should abandon the language of the necessity requirement altogether.

There are two reasons why the apparently impressive weight of authority favouring a necessity requirement is weaker than it appears. Firstly, the necessity requirement forms no part of the *ratio* of any case. I have cited many judgments invoking it, but none offer a reasoned defence of the requirement after considering its merits.^10^ This might be surprising. But consider a different element of self-defence. For well over 100 years, the orthodox position was that a defendant claiming self-defence could rely on a mistaken belief as to the facts only if that mistaken belief was held on reasonable grounds.^11^ But the merits of this *reasonable belief requirement* were not considered directly by any appellate court until 1982.^12^ Once the requirement was finally questioned, it was then repeatedly contested in the highest courts, and ultimately abandoned.^13^ Today the necessity requirement is in a similar position to the reasonable belief requirement prior to 1982. It is repeated as the orthodox legal position without an appellate court ever considering its merits.

The second reason why the authoritative support for a necessity requirement is weaker than it appears is that the authorities which refer to a necessity requirement do so in different and incompatible ways. I survey these differences in section 2. It follows that, even on the highest view of the authorities, the law does not contain a single necessity requirement supported by a great weight of authority. Rather, it contains several competing necessity requirements, each backed by much slenderer authority.

Together, these reasons destabilise the apparently strong authority upholding a necessity requirement in English law. But *should* the law contain a necessity requirement? Given the several competing formulations, to defend ‘the necessity requirement’ requires selecting one to defend. In section 3, I argue that the most legally defensible formulations do not in fact make necessity a requirement of self-defence at all, while formulations which specify a genuine requirement have little legal support and minimal effect. Worse still, that (minimal) effect is regrettable. I therefore propose abandoning the language of a necessity requirement altogether.

Abandoning the necessity requirement might sound radical. But it is not. For I am not claiming that the necessity (or otherwise) of defensive force is *irrelevant* to its lawfulness. Defensive force must still be *reasonable*, and its necessity is an important *factor* bearing on its reasonableness. What I am claiming, rather, is that the necessity of defensive force is not and should not be a *necessary condition*—a *requirement*—of reasonableness and hence lawfulness. In suitable cases, *unnecessary* defensive force can yet be reasonable and therefore lawful. (By analogy: verbal agreement is an important *factor* bearing on the existence of valid consent but is not a *necessary condition* of valid consent.) This possibility is sometimes overlooked because ‘necessary’ and ‘reasonable’ are treated as synonyms. But, as I explain in section 3, the notion of necessity is stricter than the notion of reasonableness. If the true test of lawful defensive force is reasonableness, as I contend, then courts should not reformulate this test in terms of necessity.

If I am right that the authorities contain many substantively different formulations of the necessity requirement, then the courts should, as a minimum, attempt to specify more precisely which formulation should prevail. But what if I am right that the necessity requirement ought to be abandoned? Are courts at liberty to do so, given the litany of authorities cited above? In section 4, I claim that they may do so, and indeed that Parliament has already, if implicitly, acknowledged that necessity is but one (unnecessary) reasonableness factor, rather than a necessary condition of lawful self-defence. Nonetheless, clearer codification would helpfully clarify the roles of reasonableness, necessity and proportionality in evaluating the lawfulness of self-defence.

## 2. Disambiguating Different Necessity Requirements

Imagine that C is about to shoot D. D can avoid being shot only by:

*Necessary*: Shooting C first.

*Unnecessary*: Either (i) shooting C first or else (ii) tasering C.

In *Necessary*, it is necessary for D to shoot C. In *Unnecessary*, it is not, because D had another, gentler, defensive option. These cases illustrate the comparative structure of necessity judgments: they involve weighing potential defensive options against each other. (By contrast, *proportionality* judgments weigh the defensive action against the attacker’s threat.)

But necessity judgments are ambiguous. Perhaps it was not *absolutely* necessary *to shoot* C as judged from an *impartial perspective*, but it was *reasonably* necessary *to use force* against C as judged from *the defendant’s perspective*. Which is the relevant test? Generalised, we can ask of any necessity judgment: *How* necessary? *What* must be necessary? From what *perspective* is necessity judged? This section demonstrates how different authorities have offered substantively different and incompatible answers to these questions when formulating ‘the necessity requirement’. It follows that, far from endorsing a single necessity requirement, the authorities actually endorse many substantively different requirements.

### A. How Necessary?

The first difference between different formulations of the necessity requirement concerns *how* necessary it must be to use defensive force.^14^ While some authorities refer to ‘necessity’, *simpliciter*,^15^ between others we can distinguish two views:

*Strict necessity*: Defensive force is lawful only if it was *strictly* necessary.^16^

*Reasonable necessity*: Defensive force is lawful only if it was *reasonably* necessary.^17^

The difference is significant. Consider an analogy. In a criminal trial, the prosecution must prove the defendant’s guilt to the criminal standard of proof.^18^ This requires a jury to be ‘sure’ of guilt. Directing juries on the standard of proof in any terms other than being ‘sure’ is likely to constitute a misdirection.^19^ It would be an obvious misdirection to direct a jury that they must be ‘*reasonably* sure’ of guilt. A jury might (rightly) think that being ‘reasonably’ sure was compatible with harbouring *reasonable doubts*. In other words, being *unsure*. Analogously, directing a jury that defensive force must be ‘reasonably necessary’ is very different from directing a jury that defensive force must be ‘necessary’, *simpliciter*. So we must choose between these formulations.

*Strict necessity* is *too* strict. Consider:

*Choice*: C is about to punch D. D can choose between two equally effective defensive options: (i) punching or (ii) kicking C. Punching is slightly more forceful than kicking.^20^

Punching is not strictly necessary, given the slightly gentler alternative of kicking. But denying self-defence on this basis, should D punch C, would make the law far too demanding. Nor is it how the law works in practice.^21^

That leaves *reasonable necessity*. But making sense of *reasonable necessity* is not straightforward. For ‘necessary’ is not a gradable concept. It does not come in degrees. Something is either necessary or it is not.^22^ In standard philosophical usage, ‘necessary’ means *always* the case, as distinguished from merely *possible*.^23^ A ‘necessary condition’ means just what it says. With this in mind, ‘*How* necessary?’ sounds like a malformed question and ‘*Reasonably* necessary’ a malformed answer.

One solution to this problem is to interpret ‘reasonably necessary’ as ‘reasonable *or* necessary’. This is the formulation offered by one leading textbook.^24^ As Sangero writes,

the requirement of necessity should not be interpreted in a strict and absurd fashion such that if the actor had another alternative way to repel the aggressor this would suffice to negate the existence of necessity. There may be situations in which the actor has several possible ways of action … [If D does not choose] the most moderate way … [but rather] *chooses another reasonable way*, his act is likely to comply with the requirement of necessity.^25^

The problem with this interpretation is that judging whether an action is reasonable is not the same as judging whether it is necessary. Consider:

*Burglary*: D catches C burgling his home. C is clearly unarmed, and much weaker than D, but will not leave. D picks up C and throws him out of the front door, causing scratches and bruising. D could have waited until C left of his own accord, or else removed him more delicately.

It is perfectly coherent to judge D’s use of force to be unnecessary—he had alternative, gentler, defensive options—yet reasonable. The standard of necessity is more demanding than the standard of reasonableness.^26^ If the necessity requirement only required D’s actions to be *either* necessary *or* reasonable, necessity would no longer be a *necessary condition* of lawful self-defence. The true test would be reasonableness.^27^

A different way to make sense of ‘reasonably necessary’ is to interpret ‘reasonably’ as *qualifying* ‘necessary’. If it was strictly necessary for the defendant to use (say) 1000 newtons of defensive force, then it may be *reasonably* necessary to use a degree of force which exceeds but is *sufficiently close* to that strictly necessary amount (say, 1500 newtons).^28^ But the difficulty with this interpretation is identifying what counts as ‘sufficiently close’. If only a narrow margin of error is permitted, it is likely to deny self-defence in worthy cases like *Burglary*. But if a wide margin of error is permitted, such as only forbidding force *grossly* exceeding the necessary amount, it would render the necessity requirement functionally redundant, for such grossly excessive force would be unreasonable even in the absence of a necessity requirement. Once again, it would effectively abandon the necessity requirement in favour of a generic reasonableness requirement which incorporated considerations of necessity.

Interpreting *reasonable necessity* as either ‘reasonable *or* necessary’ or ‘*sufficiently close* to strictly necessary’ is effectively to abandon a genuine necessity requirement in favour of necessity being just one factor going towards reasonableness. But there is another way to make sense of ‘reasonably necessary’. To see this, consider a problem with *strict necessity*: in one sense, defensive force is *never* necessary. Defendants always have at least one alternative to using defensive force. They could *submit* to the threat. Because submission is always an option, there is a sense in which defensive force is never strictly necessary. But this interpretation of *strict necessity* would make all defensive force unlawful. It would make the necessity requirement ‘strict and absurd’. Noticing this problem suggests a way to make sense of degrees of necessity even though the concept of necessity itself is not gradable.

Here is the thought: whether an action is necessary is assessed relative to some *end*.^29^ More actions are necessary to achieve a complex end than a narrow end. More soldiers are necessary to win a war than a skirmish. More musical notes are necessary to play a concerto than a chord. Likewise, more (and stronger) defensive actions are necessary to achieve the end of *not conceding an inch* (which might require aggressive physical measures), while fewer (and weaker) defensive actions are necessary to achieve the end of *avoiding attack* (which might require only retreat). We can therefore make sense of *reasonable necessity* as meaning ‘Necessary relative to a wider range of ends’ and *strict necessity* as ‘Necessary relative to a narrower range of ends’.^30^ Necessity *judgments* remain binary: for any given end, defensive force is necessary or not. But the necessity *requirement* can vary in strength by altering the range of ends against which necessity is judged.

This thought is obliquely reflected in the authorities, which often specify the end to which defensive force must be necessary. And different authorities offer different formulations. Many claim that force must be necessary for the defendant ‘to defend himself’. They imply:

*The end of self-defence*: Force must be necessary for D to defend themselves.^31^

(*Mutatis mutandis* for defence of another, defence of property, prevention of crime.)^32^  *The end of self-defence* is clearly more generous than the end of *doing anything else*. It does not demand submission: it permits defence. But it remains strict. For defence can often be achieved without force. *Avoiding* a threat is equally or more defensively effective than *repelling* it. It follows that *the end of self-defence* implies a *duty to retreat*. As Andrew Ashworth explains, ‘when an individual’s purpose in a threatening situation is to save himself from injury or death, it cannot be necessary for him to inflict harm on his assailant if there is a safe avenue of withdrawal open to him’.^33^

English law did historically contain a duty to retreat.^34^ But the duty to retreat was rejected by the courts,^35^ and ultimately Parliament, which clarified that: ‘[The possibility of D retreating] is to be considered (so far as relevant) as a factor to be taken into account [when judging the reasonableness of the force used], rather than as giving rise to a duty to retreat.’^36^

If defensive force was lawful only if necessary to *the end of self-defence*, this would entail a duty to retreat. But as lawful self-defence does *not* require retreat, it therefore does *not* require force to be necessary to *the end of self-defence*.

To which other end can we index the necessity requirement? First consider why English law rejected the duty to retreat. It did so because a duty to retreat inappropriately disempowers reasonable defensive actions. Consider:

*Threat*: C and D are both in the same pub. C threatens imminently to attack D unless D leaves. D knows that C will not attack if D leaves. But, instead of leaving, D responds by forcefully pushing away C.

A duty to retreat forces D to choose between relinquishing their civil rights (to remain *in situ*) or else being criminally liable for defending those rights. This was considered unfair. It was considered preferable that such citizens were afforded a defence if they used defensive force to vindicate their liberties, providing they did so in a reasonable way. This suggests a natural alternative to *the end of self-defence*, namely:

*The end of rights vindication*: Defensive force must be necessary to vindicate the defendant’s civil rights.^37^

Framing the necessity requirement in this way expands the availability of lawful self-defence to cover cases like *Threat* without abandoning necessity’s strict literal meaning. It makes sense of talk of ‘reasonable necessity’ without retreating to ‘reasonable *or* necessary’. It accommodates the absence of a duty to retreat in English law. And it finds implicit authoritative support in *Field*, a case with facts similar to *Threat*, in which the Court of Appeal permitted self-defence.^38^ As one textbook puts the point: ‘The question of sufficient necessity to use force should normally take, as its baseline, D’s right to the peaceful enjoyment of her autonomous choices.’^39^ If this is right, the necessity requirement does not require force to be necessary for self-defence. It is sufficient that force was necessary to vindicate one’s civil rights.

Interpreting the necessity requirement relative to *the end of rights vindication* undeniably weakens the necessity requirement, relative to *the end of self-defence*. We have a very wide range of civil rights. Many are trivial. If the vindication of *any* such rights qualified as a relevant end of the necessity requirement, then it would make the requirement very weak indeed.^40^ It would rule out self-defence only when the defendant used force to protect no legitimate interest whatsoever.^41^ If this is the law, then the necessity ‘requirement’ all but shades into one factor going towards reasonableness.

### B. What Must Be Necessary?

A second difference between different formulations of the necessity requirement concerns *what it is* that must be necessary; the object of necessity claims.

#### (i) Is force necessary?

The most popular formulation is that it must be necessary *to use force*.^42^ But it is controversial whether self-defence is available only in relation to *prima facie* offending involving the use of force. Call this the *force requirement*.^43^ If self-defence contains the force requirement, then it would be unremarkable for the necessity requirement to follow suit and to demand that it was necessary to use force. But if self-defence does not contain the force requirement, then this would be bizarre, given that, in fact and in law, force was not (otherwise) necessary.

The force requirement was endorsed by the Divisional Court in *Blake*. The defendant protested the Gulf War, which he believed to be illegal, by writing a biblical quotation upon a concrete pillar with marker pen.^44^ The court rejected the defence of prevention of crime on the basis that this defence ‘is only available in relevant crimes committed by the use of force’ and Blake’s graffiti did not involve force. That self-defence contains the force requirement was subsequently regarded as ‘conventional’ by the Court of Appeal,^45^ and is presupposed by the relevant statutes, which are framed in terms of the use of force, specifically.^46^ The force requirement has also been defended by the Law Commission.^47^

Against the force requirement, JC Smith points out its potential absurdity: ‘if Mr. Blake had taken a hammer and chisel and cut the letters into the concrete he might have had a defence; but as he merely wrote them with a felt pen, he did not!’^48^ Generalising the point, one textbook claims that ‘if D can justify an act of force as a response to particular circumstances, *a fortiori* justification should be accorded to a lesser, non-forceful response to the same situation’.^49^ This view has found favour in the Divisional Court, albeit *obiter*, in *Oraki*.^50^

The Court of Appeal has avoided the most absurd implications of the force requirement by watering down the meaning of ‘force’ to mean any application of energy whatsoever.^51^ But this does not address truly non-forceful examples, like driving offences involving retreat (eg speeding or running a red light) rather than forceful attack. At any rate, the courts have never offered a *principled* defence of the force requirement. For these reasons, it is arguable that the courts should reject the force requirement.^52^

Whichever view we prefer, however, we face a further interpretative question. If force is necessary, then *how much*? And if force is not necessary, then *what* must be?

#### (ii) If force is necessary, then how much force is necessary?

Assume for now that the law contains the force requirement. Two rival views remain as to *how much* force must be necessary:

*Some force*: It was necessary to use *some* force (versus none).^53^

*Degree of force*: It was necessary to use the *degree* of force which D used.^54^

The thought motivating *some force* is that once it is established that at least some force was necessary, as a first threshold, then questions as to the appropriate degree of force are more appropriately discussed under the rubric of proportionality or (other factors affecting) reasonableness.^55^

This thought can be doubted. Philosophers of self-defence standardly distinguish necessity from proportionality in the following way. Necessity concerns a relationship between the defender’s defensive harm and alternative defensive options open to the defender. By contrast, proportionality concerns a relationship between the attacker’s threatened harm and the defender’s defensive harm.^56^ To illustrate, consider:

*Options*: C is about to punch D. D can choose between exactly three equally effective defensive options: (i) shooting, (ii) punching or (iii) pushing C.

On the standard philosophical picture, shooting is both unnecessary (given the other options) and disproportionate (too extreme); punching is unnecessary (given the lesser option of pushing) but proportionate (equal to the threat); while pushing is both necessary and proportionate. This standard picture endorses *degree of force*. What makes shooting and punching unnecessary is not that *no* force was necessary—some force *was* necessary—but rather that this *degree* of force was unnecessary. Equally plainly, this picture still distinguishes the necessity requirement from the proportionality requirement, for punching was unnecessary but proportionate.

The idea motivating *some force* was that it helped to distinguish the necessity question from the proportionality question. The standard philosophical picture undermines this motivation: we can distinguish the two questions while endorsing *degree of force*. Does it follow that *some force* is mistaken?

Not necessarily. Philosophical theses do not translate directly into legal rules. We often better achieve our underlying aims through indirect rules (eg speed limits may cause safer driving than a rule directly commanding safe driving). The standard philosophical picture says that defensive force is morally permissible only if the *degree of force* was necessary. But it does not automatically follow that the law’s necessity requirement does or should duplicate that moral demand.

Remember: I do not dispute that the necessity to use the *degree of force* used is an important factor which bears on the reasonableness and hence lawfulness of any defensive force. Everyone agrees that, in *Options*, shooting would be unlawful. *Degree of force* says that this is because it violated the necessity requirement. *Some force* says that it does not violate the necessity requirement. But such force remains unreasonable, for the (distinct) reason that it is *disproportionate*. They get to the same answer, just via different routes. Of course, if *all* unnecessary degrees of force were automatically unreasonable, this would be a distinction without a difference. But that is not the case. Consider:

*Burglary 2*: D catches C burgling his home. C is clearly unarmed, and much weaker than D, but will not leave. D picks up C and throws him out of the front door, causing scratches and bruising. D could have evicted C by using slightly less force.

D’s degree of force was unnecessary because lesser force could have achieved the same end. According to *degree of force*, it would therefore be unlawful. But this seems too harsh. Unlike shooting in *Options*, this unnecessary degree of force still seems reasonable. We cannot reasonably expect people to escort burglars out of their houses using the minimum possible force. Even if D’s conduct is impermissible under some ideal moral theory, it would be astonishingly harsh on defenders to withhold self-defence on this basis. This is reflected in section 76 of the Criminal Justice and Immigration Act (CJIA), which says that ‘a person acting for a legitimate purpose may not be able to weigh to a nicety the exact measure of any necessary action’.^57^ This seems to envisage that an *unnecessary* degree of force could yet be *reasonable* in cases like *Burglary 2*.^58^ And this implies that the better interpretation of the necessity requirement is that it requires *some force* to be necessary, not the *degree of force* used.

Of course, this choice—between *some force* and *degree of force*—assumes that the law contains a force requirement. What if it does not?

#### (iii) If force is not necessary, then what is?

While most cases claim that it must be necessary *to use force*, other cases are more precise. They suggest that what must be necessary was the *specific action* taken by the defendant: actions like resorting to a gun,^59^ detaining the attacker^60^ or even going on to a football pitch.^61^ But this *specific action view* is *too* specific. Consider:

*Options 2*: C is about to punch D. D can choose between exactly two equally effective defensive options: (i) punching or (ii) kicking C. Punching and kicking are equally forceful.

The specific action of punching is unnecessary because D could instead kick. And the specific action of kicking is unnecessary because D could instead punch. The *specific action view* implausibly concludes that both actions are unnecessary and hence unlawful. That cannot be right. Where defendants have a disjunctive *set* of defensive actions (A *or* B *or* C, etc), we do not want the mere existence of other possible actions to preclude lawful self-defence.^62^

The necessity requirement should not require defendants to take *one specific* necessary action. It should only require defendants to take *a* necessary action. One way to make sense of this idea is to say that what must be necessary is that the defendant’s action *fell within the set of actions, the taking of at least one of which was necessary*. That is: if D can take action A or B or C, then so long as action A was necessary *absent* actions B and C, and likewise for B and C, then D’s chosen action would count as necessary. Call this the *set view*. In *Options 2*, punching would have been necessary if kicking were not an option, and *vice versa*. Hence, the *set view* appropriately judges both punching and kicking to be necessary in the relevant sense.

Unfortunately, the *set view* fatally undermines necessity judgments. Consider:

*Options 3*: C is about to shoot D. D can choose between exactly two equally effective defensive options: (i) punching or (ii) shooting C.

There is a disjunctive set of actions (punching C, shooting C), where each action would be necessary absent the other. The *set view* therefore deems both punching and shooting to be necessary. But this cannot be right. Shooting when punching was equally effective is a paradigmatic example of unnecessary defensive force. (It is, in substance, the case I first labelled *Unnecessary*.) More generally, *every* defensive action would be necessary *absent other options*. The whole point of necessity judgments is to compare defensive actions against other possible defensive options. Yet the *set view* prohibits precisely such comparisons.

We want to say that punching and kicking are both necessary in *Options 2*, but that only punching and not shooting are necessary in *Options 3*. The reason, we might think, is that punching and kicking are equally justifiable, while punching and shooting are not. This can be achieved with the *equally justified set view*, which is as per the *set view*, but the disjunctive set of defensive options comprises only actions which are equally as justified as the defendant’s action. But consider:

*Options 4*: as per *Options 2* (punching or kicking), but punching is marginally more harmful than kicking.

Punching is now (marginally) less justified than kicking. Punching therefore falls outside the set of equally justified actions and is therefore considered unnecessary on the *equally justified set view*. But this returns us to the problems associated with *degree of force*. The necessity requirement should not consider defensive force to be unnecessary (hence unlawful) merely because there was a *marginally* preferable alternative option.^63^

A plausible necessity requirement must permit non-optimal defensive actions whilst forbidding actions which are significantly worse than available alternatives. This implies that what must be necessary is that the defendant’s action fell not within a set of *equally* justified actions, but rather a set of *similarly* justified actions, the taking of at least one of which was necessary. Call this *reasonable set view*.

The *reasonable set view* solves the problems of the previous interpretations. Unlike both *some force* and *degree of force*, it permits non-forceful defensive actions (such as assault by threats or running a red light). Unlike the *specific action view* and the *equally justified set view*, it permits non-optimal defensive actions. And unlike the *set view*, it forbids highly sub-optimal defensive actions. The price of success, however, is significantly eroding the distinctive nature of necessity judgments. Consider:

*Options 5*: C is about to punch D. D can choose between exactly three equally effective defensive options: (i) punching C, (ii) causing C to suffer a mild allergic reaction or (iii) threatening to kill C (without intending to follow through).

I do not know whether a jury would deem these options to be sufficiently similarly justified. But what is clear is that this enquiry looks quite different from ‘classic’ necessity enquiries. We are no longer asking whether D’s *force* or *action* was necessary, but rather whether their action fell within a reasonable range. And this is functionally very similar to abandoning the necessity requirement altogether and replacing it with a generic assessment of reasonableness; of turning necessity into just one factor going to reasonableness.

### C. Necessary in Fact or Belief?

The third and final difference between different formulations of the necessity requirement concerns the perspective from which we judge necessity.

Some authorities judge necessity from an objective perspective. They claim that defensive force must be ‘necessary’, *simpliciter*.^64^ Other authorities judge necessity from a subjective perspective. They claim that the defendant must ‘honestly *believe* … that it was necessary to defend himself’.^65^ Call these views, respectively:

*Factual necessity*: Defensive force is lawful only if it was *factually* necessary.

*Believed necessity*: Defensive force is lawful only if *D believed* that it was necessary.

(I refer to ‘defensive force’ for convenience, not because I endorse the force requirement.) To illustrate the difference, consider:

*Mistake*: D hears C approaching behind her. Believing she is about to be attacked, D hits C. In fact, C was harmlessly jogging.

D (subjectively) believed defensive force to be necessary despite it not being (objectively) factually necessary. These different formulations of the necessity requirement seemingly result in different outcomes.

But this appearance is misleading. As noted earlier, where defendants are mistaken as to the relevant facts, their conduct is judged relative to the facts which the defendant (subjectively) believed to exist, not the facts which (objectively) existed. It follows that *factual necessity* actually amounts to:

*(Belief-relative) factual necessity*: Defensive force is lawful only if D *believed in facts*, *which, if true, would make force factually* necessary.^66^

In other words, even if the necessity of using force is judged objectively, the *relevant facts* are judged subjectively.^67^ This brings the two perspectives closer together. In *Mistake*, D (subjectively) believed that force was necessary. And D believed this because D (subjectively) believed in facts (that C was about to attack her) which, if true, would (objectively) make it factually necessary to use force.^68^  *(Belief-relative) factual necessity* and *believed necessity* therefore result in the same outcome in *Mistake*.^69^

But while *(belief-relative) factual necessity* is closer to *believed necessity* than was *factual necessity*, they are not identical. For a defendant can be mistaken that defensive force is necessary without being mistaken about the underlying facts. Consider:

*Mistake 2*: D sees C approach. Correctly believing that C plans to snatch D’s phone, D punches C. D failed to consider that he could have avoided the theft simply by putting his phone in his pocket.

D believed his defensive force to be necessary. He satisfies *believed necessity*. This belief was mistaken. He could have avoided theft simply by hiding his phone.^70^ Crucially, however, his mistake derived from his failure to reason about alternative options—about the necessity of defensive force *per se*—rather than a failure to perceive the relevant facts. He did not believe in any facts which, if true, would have made defensive force necessary.^71^ He therefore fails to satisfy *(belief-relative) factual necessity*.

*(Belief-relative) factual necessity* permits reliance on mistaken self-defence only where D was mistaken about primary facts, as in *Mistake*. By contrast, *believed necessity* permits reliance on mistaken self-defence even where D was mistaken about the demands of necessity itself, as in *Mistake 2*. *Believed necessity* is therefore more lenient than *(belief-relative) factual necessity*. They imply substantively different necessity requirements.^72^

Which formulation is to be preferred? On the one hand, defendants cannot typically rely on mistakes about the very evaluative standards by which they are to be judged. Elsewhere in the criminal law, it is irrelevant that defendants believe their conduct to be reasonable or honest. What counts is whether their conduct was *in fact* reasonable or honest (albeit judged relative to whatever facts they (perhaps mistakenly) believed to exist).^73^ This general approach supports *(belief-relative) factual necessity*.

On the other hand, proponents of the necessity requirement do not claim it to be a *sufficient* condition of lawful self-defence. Even necessary defensive force must still be (objectively) reasonable, which includes considerations of proportionality.^74^ Given this further control mechanism, demanding even *(belief-relative) factual necessity* may be to demand too much. If defendants like D in *Mistake 2* are (at least sometimes) worthy of self-defence—*providing their response was reasonable*—then *believed necessity* is to be preferred. This may well be appropriate. It would be harsh for self-defence to fail merely because the defendant, who acted reasonably, perhaps in the heat of the moment, failed to consider the availability of some alternative option. And this view is supported by Parliament, which clarified that if a defendant ‘honestly and instinctively *thought* [their actions were] necessary’—ie *believed necessity*—this ‘constitutes strong evidence that only reasonable action was taken’.^75^

## 3. Against the Necessity Requirement

I have distinguished several different versions of the necessity requirement. Some are substantially more demanding than others (Table 1).

**Table 1.** Disambiguations of ‘the’ necessity requirement

	Less demanding	More demanding
***How* necessary?**	Reasonable or necessary; Sufficiently close to necessary	Strictly necessary
**(Strictly) Necessary *to What End*?**	Vindicating civil rights	Self-defence
***What* Must be Necessary?** (if force is required)	Some force	The degree of force used
***What* Must be Necessary?** (if force is not required)	D’s action fell within the set of similarly justified actions, the taking of at least one of which was necessary	D’s precise action
**Necessary *in fact or belief?***	Believed to be necessary	Factually necessary, relative to D’s beliefs as to the primary facts

At one extreme, the necessity requirement would demand that D believe *in facts* which, if true, would render their *precise action strictly* necessary to the end of *self-defence*. At the other extreme, the necessity requirement would demand only that D *believe* that *their action fell within a set of similarly justified actions, the taking of at least one of which* was necessary *or reasonable* to *vindicate any right*. These are very different propositions.

The most demanding versions of the necessity requirement are untenable as a matter of positive law. The law simply does not require defenders’ actions to be strictly necessary to the end of self-defence, for that is incompatible with the absence of a duty to retreat. Nor does it demand that defenders’ precise degree of force, or their precise action, was strictly necessary, for that would be incompatible with the leeway to take (somewhat) suboptimal actions implicitly acknowledged in the decided cases and Parliament’s suggestion that defenders may act lawfully despite ‘not be[ing] able to weigh to a nicety the exact measure of any [“]necessary[”] action’.^76^ Similarly, Parliament’s suggestion that reasonableness (in part) depends on whether defenders ‘honestly and instinctively *thought* [their actions were] necessary’ is not easily compatible with necessity requirements which claim that force must be *factually* necessary (on the facts which D believed to exist).^77^

By contrast, the least demanding versions of the necessity requirement identify uncontroversial legal propositions. It is clearly true that lawful defensive force must be ‘necessary *or* reasonable’, for the reasonableness requirement—unlike the necessity requirement—is not in dispute. Likewise, the requirement to take an action which falls within a set of *similarly* justified actions, the taking of at least one of which was necessary, functionally amounts to a (mere) reasonableness requirement. The only problem with these weaker versions of the necessity requirement is that they are misleadingly named. For they do not truly make necessity a *requirement*, a necessary condition, of self-defence. They merely highlight that defensive force must be *reasonable*, and that necessity is one factor (among others) going to reasonableness. And this was never in dispute.

Between those extremes, however, lie versions of the necessity requirement which are neither incompatible with the positive law nor reducible to a mere reasonableness factor. The law *could* require defenders to *believe* (in facts such) that *some force* was *strictly* necessary to the end of *vindicating their civil rights*. But no authority *does* so in these exact terms. This version of the necessity requirement is highly complex. And it has minimal legal effect: it prohibits virtually no instance of defensive force not already prohibited by a much simpler standard of reasonableness. These factors should give us pause as to whether the law ought to contain a necessity requirement at all.

Worse still, even the minimal effect of such a necessity requirement would be regrettable. Consider two more cases:

*Mugging*: While he is out jogging, two men threaten to beat up D unless he hands over his phone. D is a professional athlete and knows that he could simply run away. But D roughly knocks them to the floor as he leaves, to dissuade any pursuit.

*Arrest*: D, a police officer, is lawfully arresting a drunken offender. The offender ineffectually swings his arms towards D. D correctly judges that the offender will continue to miss, and that D could slowly persuade the offender to calm down. However, to hasten the encounter, D instead decides to firmly restrain the offender.

These defendants knew that force was not strictly necessary to defend themselves or vindicate their rights. But should we deny them the benefit of self-defence, merely because they knew that their conduct (or any use of force) was not, strictly speaking, necessary? I think not. They acted eminently reasonably in defence of valid legal interests, in situations which could have escalated to become more serious or onerous. The law should not deny them a defence.^78^ But that would be the effect of even viable versions of the necessity requirement. For these reasons, I propose that the law abandon even these versions of the necessity requirement. It should acknowledge that necessity is but one, unnecessary, factor going to the reasonableness of any defensive force.

What makes the standard of reasonableness more appropriate than the necessity requirement? Broadly speaking, the standard of reasonableness assesses whether something is acceptable on the balance of reasons.^79^ It is a holistic standard, not restricted to a particular domain of reasons.^80^ Clearly, there are reasons not to use unnecessary force.^81^ But, in suitable cases, these reasons might be outweighed by reasons in favour of using such force. In *Mugging*, the reasons to use force (deterring pursuit) plausibly outweigh the reasons against (it being unnecessary), such that pushing the muggers was reasonable on balance.^82^ Likewise in *Arrest*: the reasons favouring force (strategically defusing and hastening a tense situation) plausibly outweigh the reasons against (non-necessity). This is why unnecessary force can yet be reasonable. Making necessity a necessary condition of reasonableness, rather than one factor in the equation, overstates the importance of reasons of necessity relative to other reasons.

The law of self-defence has already abandoned a once-central requirement: the reasonable belief requirement. Comparing the two cases only strengthens the case for abandoning the necessity requirement. Abandoning the reasonable belief requirement left no objective standard by which to judge defendants’ beliefs. It made no exception for blameworthy mistakes, such as those formed due to racial bias.^83^ By contrast, abandoning the necessity requirement leaves the rigorously objective standard of reasonableness by which to judge defendants’ actions. Abandoning the necessity requirement is therefore much easier to justify.

Of course, objections are possible. I will consider four. Firstly, even accepting that ‘reasonable’ is more permissive than ‘necessary’, it might be objected that the distinction is minimal; that judges and juries are likely to interpret these terms in much the same way. This certainly seems possible, given that judges have often treated ‘necessary’ and ‘reasonable’ interchangeably. But, for the reasons given above, necessity and reasonableness are distinct concepts. Using different terms in different cases creates a real risk that at least *some* juries will reach different conclusions depending on which word is used; that they will treat like cases unalike. (All the more so if prosecutors emphasise the literal meaning of ‘necessary’.) As noted above, asking juries to be ‘reasonably sure’ (versus ‘sure’) constitutes a misdirection on the standard of proof. Likewise, if the real evaluative test is reasonableness, as I have argued, then asking whether defenders’ actions were ‘necessary’ should constitute a misdirection on self-defence.

Secondly, abandoning the necessity requirement seemingly puts the law in conflict with the philosophical consensus that necessity is a requirement of morally justified self-defence.^84^ My response, raised above, is that we should not blindly translate philosophical theses into legal rules. Necessity as a reasonableness factor captures the moral significance of necessity in self-defence without suffering the practical downsides of enacting it as a bright-line legal rule. Further, however, the philosophical consensus in support of the necessity requirement, much like the legal consensus, is more apparent than real. For different philosophers offer strikingly different accounts of the necessity requirement’s basis and demands.^85^ Some accounts are especially ill-suited to legal enactment. For instance, one leading account explains the necessity requirement by reference to a deeper duty to rescue.^86^ Whatever the merits of that account, it is a poor fit with English law, which categorically rejects a duty to rescue outside of specific narrow categories. The philosophical consensus in favour of a *moral* necessity requirement does not justify a *legal* necessity requirement.

Thirdly, it might be objected that abandoning the necessity requirement will have collateral damage. For, while I have distinguished many different versions of the necessity requirement, I have not canvassed *every* thesis which might fly under that banner. According to one textbook,

Force cannot be justified to prevent a crime or to defend against an attack unless it was a sufficiently necessary means of doing so. This includes a causal intentional requirement, namely that D uses force *in order to* defend against attack or prevent crime, which excludes force used in revenge against previous attacks from V.^87^

This ‘causal intentional requirement’ is more simply described as the *purposive* requirement, according to which defensive force is lawful only if D acted *in order to* defend themself; only if D’s conduct was defensively motivated.^88^ The problem with this passage is that one can use force *in order to* defend oneself (the purposive requirement) without such force being (believed to be) *necessary* to defend oneself (a necessity requirement). They are distinct requirements. And this is important because, while I propose abandoning the necessity requirement, I do not propose abandoning the purposive requirement. If anything, I believe that the purposive requirement ought to be emphasised more strongly than is typically the case.^89^ So I agree that it would be a problem if, by abandoning the necessity requirement, the courts unwittingly abandoned the purposive requirement. But such collateral damage is easily avoided by simply distinguishing the two requirements.

Fourthly, and finally, one might object that judging defensive actions only by an undifferentiated standard of reasonableness is worrisomely vague. One virtue of the necessity requirement, the thought goes, is providing structure to fact finders’ evaluation of the defendant’s conduct. And abandoning the necessity requirement means abandoning that virtue. A simple reply to this objection is that the disvalue of the necessity requirement—denying a defence to those whose unnecessary-but-reasonable conduct warrants a defence—outweighs the benefit of adding structure to the evaluative exercise. But a better reply is that necessity can play an important structural role in evaluating self-defence without being a *necessary condition* of its lawfulness. As I have emphasised throughout, necessity would and should remain an important factor going to the reasonableness and hence lawfulness of self-defence. Judges can make this clear to juries in their directions: while the jury must ultimately evaluate whether the defendant used reasonable force, they should consider the extent to which the defendant’s actions were necessary as an important factor within that broader enquiry. Sensible legislation could also emphasise this point. It might even create a rebuttable *presumption* that unnecessary force is unreasonable.^90^ But it would not make necessity a *necessary condition* of lawful self-defence.

## 4. What Follows?

I have proposed abandoning the necessity requirement. But what does this mean in practice? Are courts at liberty to abandon it, notwithstanding the litany of authorities claiming that necessity is a condition of self-defence? Or is parliamentary intervention required?

I believe that courts are entitled to abandon the necessity requirement without parliamentary intervention. For, while many authorities *claim* that necessity is a necessary condition of lawful self-defence, *in practice* they usually interpret it in a way that amounts to just one reasonableness factor. Indeed, some authorities go further: they explicitly define the evaluative test for lawful self-defence solely in terms of reasonableness, without a distinct necessity requirement.^91^ Future courts may also draw on the persuasive authority of necessity-free formulations of self-defence from Law Commission proposals,^92^ foreign criminal codes^93^ and the Rome Statute of the International Criminal Court.^94^

But the strongest reason why parliamentary intervention is not required is that Parliament has *already* ‘clarified’ the law of self-defence in section 76 of the CJIA. This clarification assumed that the operative evaluative question is ‘whether the degree of force used by D … was *reasonable* in the circumstances’.^95^  *Reasonable*, not necessary. Section 76’s only action-guiding reference to necessity directs decision makers ‘that evidence of a person’s having only done what the person honestly and instinctively thought was necessary for a legitimate purpose constitutes strong evidence that only reasonable action was taken by that person for that purpose’.^96^

‘Strong evidence’ does not mean ‘necessary condition’. It means ‘an important factor in favour’. Parliament therefore seems to envision (believed) necessity as but one reasonableness factor: not a requirement, a necessary condition, of the reasonableness and lawfulness of self-defence.^97^

Admittedly, section 76 did not purport to replace the common law, only to ‘clarify’ it.^98^ If the common law contained a necessity requirement, as many authorities claim, then section 76 would not clearly override those authorities. But, once we acknowledge the frailty of the common law support for a necessity requirement, section 76 may provide sufficient textual cover for courts explicitly to abandon the language of a necessity requirement altogether.

Nevertheless, explicit statutory language to this effect would be preferable. When Parliament ‘clarified’ the law, it should have said that defensive force is lawful only if it is reasonable, and that necessity is (merely) one important factor bearing on reasonableness, and not a necessary condition of reasonableness.

## 5. Conclusion

I have argued that ‘the necessity requirement’ is not a single condition of self-defence, supported by a great weight of authority. Instead, it refers to many different possible requirements. Stronger versions are untenable as a matter of law, while viable versions would make the law worse. The dominant interpretation of the necessity requirement, in practice, is that defensive conduct must be *reasonable*, and that necessity is one factor bearing on reasonableness. This interpretation puts the law in its best light. But it does not make necessity a *requirement*, a necessary condition, of self-defence. For this reason, the law should abandon the language of a necessity requirement. It should no longer say, without clarification or caveat, that defensive force *must* be necessary to be lawful.

CMV Clarkson once pointed out that the courts have sometimes treated the defences of self-defence, duress and necessity as interchangeable, or as sub-species of the same defence.^99^ He described ‘an increasing realisation that clear demarcation between these defences is highly problematic’.^100^ His solution was to go further still, by unifying them into a single defence of ‘necessary action’. Predictably enough, I think it would be a mistake to subsume self-defence into a broader defence of ‘necessary action’. For I think that self-defence can be lawful even if unnecessary. Abandoning the necessity requirement allows us more clearly to delineate these distinct defences. For while self-defence should abandon the necessity requirement, a general defence of necessity could embrace it. As the name implies, if one hopes to rely on a defence of necessity, perhaps one’s actions must be truly necessary: strictly, literally, narrowly necessary.^101^ This is not to defend a general defence of necessity. That would take us too far beyond my topic. But rejecting the necessity requirement in self-defence at least opens up distinct space for a distinct necessity defence.^102^

## Footnotes

1 I use ‘defensive force’ as a catch-all term for both private defence (self-defence, defence of another and defence of property) and public defence (prevention of crime and lawful arrest). As is common, I will sometimes use just ‘self-defence’ as the relevant catch-all.

2 This formulation is a placeholder. I disambiguate different versions of the necessity requirement in s 2.

3
*Weston* (1879) 14 Cox CC 346, 351.

4
*Rose* (1884) 15 Cox CC 540, 541. Contemporary law reformers proposed to codify this ‘restriction that the force used is necessary’: Criminal Code Bill Commission, *Report of the Royal Commission appointed to Consider the law Relating to Indictable Offences* (London 1879) 11.

5
*Palmer (Sigismund)* [1971] AC 814, 832.

6 David Ormerod and David Perry (eds), *Blackstone’s Criminal Practice* (33rd edn, OUP 2023) A3.59: ‘were the facts (as D believed them to be) such that the use of force was necessary …?’; David Ormerod and Karl Laird, *Smith, Hogan, and Ormerod’s Criminal Law* (16th edn, OUP 2021) 401, fn 340: ‘D must have a genuine belief in … the necessity of using force’; JJ Child and others, *Simester and Sullivan’s Criminal Law* (8th edn, Hart Publishing 2022) 887: ‘it must have been sufficiently necessary for D to use some force against V to protect himself or another’.

7 German Criminal Code (StGB), §32: ‘Self-defense means defensive action *that is necessary* to avert an imminent unlawful attack on oneself or another’ (translated in Markus Dubber and Tatjana Hörnle, *Criminal Law: A Comparative Approach* (OUP 2014) 406); American Law Institute, *Model Penal Code* (1962), s 3.04: ‘the use of force upon or toward another person is justifiable when the actor *believes that such force is immediately necessary* for the purpose of protecting himself against the use of unlawful force by such other person on the present occasion.’ See also New South Wales’s Crimes Act 1900, s 418(2): ‘A person carries out conduct in self-defence if and only if the person *believes the conduct is necessary*—(a) *to defend himself*’; and Victoria’s Crimes Act 1958, s 322K(2): ‘A person carries out conduct in self-defence if—(a) the person *believes that the conduct is necessary in self-defence*’ (all emphases added). For customary international law, see the ‘Caroline test’: ‘a necessity of self-defence, instant, overwhelming, leaving no choice of means, and no moment for deliberation’: Daniel Webster, ‘Letter to Henry Stephen Fox’ (24 April 1841).

8 ‘Philosophers who work on the ethics of defensive force are nearly unanimous in claiming that the imposition of defensive harm is permissible only if it meets the necessity condition’: Jonathan Quong, *The Morality of Defensive Force* (OUP 2020) 125.

9 Boaz Sangero, *Self-Defence in Criminal Law* (Hart Publishing 2006) 143. Sangero is sufficiently confident in this view to offer the following footnote: ‘Given the fact that there is no dispute with regard to the requirement of necessity—in legislation, in case law and in legal and philosophical literature, both of the past and of the present—I will refrain from making a list of references for this subject’ (fn 612).

10 It is difficult to prove a negative—that *no* English case has ever offered a reasoned defence of the necessity requirement—but none of the (many) English cases cited in this article nor any other I have found via standard searches do so.

11
*Forster* (1825) 1 Lew 187, 188: defensive force can be justified only if D had ‘reasonable ground for believing himself to be in peril’.

12
*Albert v Lavin* [1981] 2 WLR 1070, 1076: ‘there appears to be no reported instance of a man being convicted of an assault … when he acted, as he believed, in self-defence, but the belief was held to be unreasonable. However, nearly all the authorities, when considering self-defence, require that a mistaken belief must be reasonable.’ This consideration of the point was likely spurred by ‘subjectivist’ academic criticism: Hodgson J (1077) cited ‘vigorous’ criticism from the leading textbooks of the day: Glanville Williams’ *Criminal Law*; Kenny’s *Outlines*; *Russell on Crime*; *Smith and Hogan*.

13
*Kimber* [1983] 1 WLR 1118; *Williams (Gladstone)* (1983) 78 Cr App R 276; *Beckford* [1988] AC 130. These cases in turn prompted a wealth of further academic discussion: Jeremy Horder, ‘Cognition, Emotion and Criminal Responsibility’ (1990) 106 LQR 469; AP Simester, ‘Mistakes in Defence’ (1992) 12 OJLS 295. The rejection of a reasonable belief requirement was put on a statutory footing by the Criminal Justice and Immigration Act 2008 (CJIA), s 76(3)–(4).

14 I assume for now that the object of the necessity requirement—*what* must be necessary—is the use of defensive force. I question this assumption in s 2B.

15 HHJ Martin Picton and others, *The Crown Court Compendium: Part 1* (June 2022) 18-1[1] simply asks whether ‘D believe[d] … that it was necessary to use force’.

16 Criminal Code Bill Commission (n 4) 11: defence should be available only if ‘the mischief sought to be prevented *could not* be prevented by less violent means’; *Rose* (n 4) 541: ‘the fatal shot …was *absolutely necessary* for the preservation of … life’; Suzanne Uniacke, *Permissible Killing: The Self-defence Justification of Homicide* (CUP 1994) 32: ‘The sense of “necessity” relevant to Self-Defence … is *indispensability or unavoidability*’ (all emphases added).

17
*Chisam* [1963] 47 Cr App R 130, 133: ‘There must be a *reasonable* necessity for the killing’; *Palmer* (n 5) 831: ‘a defendant may do “what is *reasonably* necessary”’; *Keane, McGrath* [2010] EWCA Crim 2514 [19]: ‘what is not unlawful is force which is reasonably necessary’; *Smith, Hogan, and Ormerod* (n 6) 401: ‘*reasonable or* necessary … to use force’; *Simester and Sullivan* (n 6) 887, 890, 892: force is ‘*sufficiently* necessary’ (emphases added).

18
*Woolmington v DPP* [1935] AC 462.

19 A ‘reminder that the prosecution has to make the jury “sure” in order to prove guilt is probably the safest course to adopt’: *Crown Court Compendium* (n 15) 5-1.

20 True, using *some* force was necessary. I consider different interpretations as to *what* must be necessary in s 2B, including variations of this case.

21 ‘[A] person acting for a legitimate purpose may not be able to weigh to a nicety the exact measure of any necessary action’: CJIA, s 76(7)(a). ‘Legitimate purpose’ is defined in s 76(10)(a) as self-defence, defence of property, prevention of crime or lawful arrest.

22 The *Oxford English Dictionary* defines ‘necessary’ as ‘Indispensable, vital, essential; requisite’.

23 James Garson, ‘Modal Logic’ in Edward N Zalta and Uri Nodelman (eds), *The Stanford Encyclopedia of Philosophy* (Spring edn, 2023). Philosophers do distinguish different types of necessity, eg nomic necessity (mandated by natural laws) logical necessity (mandated by logic), metaphysical necessity (mandated in some deeper way), and so on: Boris Kment, ‘Varieties of Modality’ in Edward N Zalta (ed), *The Stanford Encyclopedia of Philosophy* (Spring edn, 2021). But those distinctions can be safely ignored in this context.

24
*Smith, Hogan, and Ormerod* (n 6) 401.

25 Sangero (n 9) 145–6 (emphasis added). Sangero’s initial point concerns overdetermination: the mere addition of *some* alternatives should not undermine the lawfulness of any given option. But judgments about necessity inevitably require us to judge the defender’s action against other alternatives. I discuss this problem in s 2B, below.

26 I defend this claim in more detail in s 3.

27 Art 2.2 of the European Convention on Human Rights permits causing death only if doing so ‘is no more than *absolutely necessary* … in defence of any person from unlawful violence’ (*inter alia*). The language of ‘absolutely necessary’ seems definitive. Yet, in practice, ‘the [European Court of Human Rights] has consistently held that the reasonableness limb of self-defence … is compatible with the article 2.2 requirement of “absolute necessity”’: *R (Collins) v Secretary of State for Justice* [2016] EWHC 33 (Admin) [63]. Whatever the pragmatic benefits of such an interpretation, it does not faithfully reflect the language of ‘absolute necessity’.

28 See eg *Simester and Sullivan* (n 6) 887, 890, 892, which talks of force being ‘*sufficiently* necessary’. Here, I assume that *what* must be necessary is the defendants’ degree of force. I question this assumption in s 2B.

29 Uniacke (n 16) 33: ‘in explaining why a particular degree of force is necessary we refer to some aim, purpose, or end for which, or in the achievement of which, this force is indispensable or unavoidable’.

30 (But not so narrow as the end of *doing anything else*, for that would include mere submission.)

31 The phrase ‘to defend himself’ is the formulation used in *Weston* (n 3) 351, *Palmer* (n 5), 831, *Rashford* [2005] EWCA Crim 3377 [19], *Dewar v DPP* [2010] EWHC 1050 (Admin) [7] and *Yaman* [2012] EWCA Crim 1075 [31]. See also Andrew Ashworth, ‘Self-Defence and the Right to Life’ (1975) 34 CLJ 282, 285: ‘necessary for the purpose of self-defence’.

32
*Faraj* [2007] EWCA Crim 1033; [2007] 2 Cr App R 25 [8]: ‘to protect his property’; *Dewar* (n 31) [9]: ‘to defend his son from attack’; *Smith, Hogan, and Ormerod* (n 6) 417: ‘to prevent the crime or effect the arrest’ (in relation to s 3 of the Criminal Law Act 1967 (CLA)); *Simester and Sullivan* (n 6) 887: ‘to protect himself or another, or in the prevention of crime’.

33 Ashworth (n 31) 284. See also Fiona Leverick, *Killing in Self-Defence* (OUP 2006) 5: ‘if an attack could have been avoided without resorting to force (by, for example, taking an opportunity to escape the aggressor), then any force used in self-defence was not necessary.’

34
*Julien* [1969] 1 WLR 839. Householders defending their homes were excepted from this duty ‘for that would be giving up his house to his adversary’: *Hussey* (1924) 18 Cr App R 160, 161. Rather like the reasonable belief requirement, Leverick points out that this duty was not subject to extended judicial analysis until the 20th century: Leverick (n 33) 70.

35
*McInnes* [1971] 1 WLR 1600; *Bird* [1985] 1 WLR 816. The English courts were significantly influenced by the position in the United States and Australia. Oliver Wendell Holmes of the US Supreme Court claimed that failure to retreat was merely ‘a circumstance to be considered with all the others in order to determine whether the defendant went farther than he was justified in doing’: *Brown v United States* 256 US 335 (1921) 343. The Australian High Court later held that ‘to retreat before employing force is no longer to be treated as an independent and imperative condition if a plea of self-defence is to be made out … whether a retreat could and should have been made is an element for the jury to consider as entering into the reasonableness of the defendant’s conduct’: *Howe* (1958) 100 CLR 448, 463.

36 CJIA, s 76(6A).

37 By ‘rights’, I mean to include both Hohfeldian claim rights and privileges/liberties. For an introduction, see Ori Herstein, ‘Legal Rights’ in Edward N Zalta and Uri Nodelman (eds), *The Stanford Encyclopedia of Philosophy* (Winter edn, 2023).

38 This is loosely based upon *Field* [1972] Crim LR 435.

39
*Simester and Sullivan* (n 6) 892.

40 The courts could specify a limited list (or type) of qualifying rights. But this adds yet another (complex) interpretative question on top of all the others raised in this article.

41 Note that trivial rights infringements would still only justify trivial degrees of defensive force, as more force would be unreasonable for the (distinct) reason that it was *disproportionate*. The *necessity requirement* (interpreted thus) would not object to such force, but *proportionality* would.

42 See eg the Explanatory Note to CJIA, s 76: ‘necessary to use force’; *Yaman* (n 31) [31]: ‘it was necessary to use force’; *Smith, Hogan, and Ormerod* (n 6) 401: ‘necessary for him to use force’, ‘the necessity of using force’. Not all cases identify the object of necessity claims: *Palmer* (n 5) talks merely in terms of ‘what is reasonably necessary’ without further elaboration.

43 Some authorities have permitted self-defence in cases where D merely *threatened* to use force or *prepared* to do so: *Cousins* [1982] QB 526, 530; *Attorney General’s Reference (No 2 of 1983)* [1984] QB 456. The force requirement should be read expansively to include these, as in *Faraj* (n 32) [8]: ‘did the defendant honestly believe that he needed to use *the threat* of force’? (Emphasis added).The Law Commission’s *Draft Criminal Code* added a clause that ‘A person does not commit an offence by doing an act immediately preparatory to the use of such force’: Law Commission, *A Criminal Code for England and Wales: Volume 1* (Law Com No 177, 1989) cl 44(5).

44
*Blake* [1993] Crim LR 586 (DC). The report only mentions a defence under s 3 of the CLA, but only prevention of crime makes sense on the context of the facts. The CLA was assumed to share the same normative framework as common law self-defence by the CJIA.

45
*Riddell* [2017] EWCA Crim 413, [2017] 1 WLR 3593 [41]. Based on the premise that self-defence contains a force requirement, the Court of Appeal has reasoned at some length as to whether it constitutes a use of ‘force’ to attach oneself to a tractor and (separately) to drive a car at a person: *Bayer* [2003] EWHC 2567, [2004] 1 WLR 2856 [25]; *Riddell* (idem) [41]–[43].

46 CLA, s 3: ‘A person may use *such force* as is reasonable in the circumstances in the prevention of crime’; CJIA, s 76(1) refers to ‘the question [of] … *whether the degree of force used* by D … was reasonable’ (emphases added).

47 Law Commission, *Legislating the Criminal Code: Offences against the Person and General Principles: A Consultation Paper* (Law Com CP No 122, 1992) 20.5. They defended the force requirement on the basis that it was familiar to the law and removing it would slow down the codification process. More substantively, they argued that ‘if the limitation to the use of force were abandoned, there would be no apparent stopping-point short of a general, and almost inevitably unelaborated, proposition that one may do any act that it is reasonable in the circumstances to do’. But this argument would equally justify limiting self-defence to actions involving, say, *severe* force. It is really a complaint against the open-textured nature of the reasonableness standard, not the set of actions which fall to be judged by that standard. In their final report, the Law Commission took the absence of adverse comments on their view expressed in the consultation paper as evidence in favour of the force requirement: (Law Com No 218, 1993) 38.2, fn 381. A more sceptical interpretation is that they should not have expected commentary on every aspect of a 140-page consultation document spanning both offences against the person and general defences, which was published only three years after the Commission’s 290-page *Draft Code* and commentaries. I offer a stronger version of their argument in n 52.

48 Commentary to *Blake* (n 44).

49
*Simester and Sullivan* (n 6) 888.

50
*Oraki* [2018] EWHC 115 Admin, [2018] 2 WLR 1725 [28].

51
*Swales v Cox* [1981] 1 QB 849, 855; *Bayer* (n 45) [25].

52 A principled defence is possible. One of the law’s paramount coordinative functions is to authoritatively resolve conflicts of interests. If all criminal proscriptions (which protect certain interests) could be defended against by invoking supervening conflicting interests which the law does not otherwise acknowledge, this would undermine the law’s coordinative function. This explains why the law has consistently refused to permit a general defence of necessity. Instead, it confines defences to a well-established and limited group, among which is self-defence. If self-defence were not restricted to the use of force, then it would begin to resemble an amorphous necessity defence. This is one reason to limit it to uses of force. Indeed, non-forceful driving offences can fall under the distinct defence of duress of circumstances, in order to reflect this distinction. While this argument has some attraction, ultimately I doubt that it can sufficiently justify the *prima facie* absurdity involved in cases like *Blake* (n 44).

53 Leverick (n 33) 5: ‘Necessity refers to the need to use defensive force *at all* … [ie] it is necessary to use *at least some force* to avoid an attack’ (emphases in original).

54 Criminal Code Bill Commission (n 4) 11: ‘the restriction that *the force used* is necessary’ (not just ‘some’ force); Ashworth (n 31): ‘the *amount* of force should have been no more than necessary’; Uniacke (n 16) 32: ‘[the necessity requirement asks] why a particular *degree* of force is necessary’; Sangero (n 9) 144: ‘[D must use a] quantitatively [necessary]… *measure* of…defensive force’ (all emphases added).

55 Leverick (n 33); *Dewar* (n 31) [9]: ‘The first stage is to decide whether there was an honest belief that *some force* was needed … The fact … that the force actually used is excessive goes to the second stage, namely consideration of whether in the circumstances the force was reasonable’ (emphasis added).

56 See, generally, Helen Frowe and Jonathan Parry, ‘Self-Defense’ in Edward N Zalta (ed), *The Stanford Encylopedia of Philosophy* (Summer edn, 2022).

57 CJIA, s 76(7)(a). ‘Legitimate purpose’ is defined in s 76(10)(a) as self-defence, defence of property, prevention of crime or lawful arrest.

58 Unfortunately, the reference to ‘necessary action’ is ambiguous. Is the claim that D may not be able to weigh the measure of an action, *including* its necessity (my interpretation), or that D may not be able to weigh the measure of an action which nonetheless *must be* necessary?

59
*Weston* (n 3) 351: ‘[Did] the prisoner *resort* … *to the gun* … in necessary self-defence[?]’; similarly, in *Rose* (n 4) 541, Lopes J asked whether ‘*the fatal shot which [the defendant] fired* was absolutely necessary’ (emphases added). While similar, this formulation problematically refers to a *consequence*—causing death—instead of an *action* taken by the defendant (on which see Sangero (n 9) 145).

60
*Faraj* (n 32) [22]: D ‘must honestly believe that he needs *to detain the suspect*’ (emphasis added).

61
*Dewar* (n 31) [8]: Did D ‘honestly believe that it was necessary *to go onto the pitch* in order to defend his son[?]’ (emphasis added). (As noted above, *Dewar* elsewhere claims that what must be necessary is the use of ‘some force’. The Crown Court seems to have denied that D believed that his *punch* was necessary, while accepting that *pulling* C might have been.) Other cases take a similar approach but refer generically to the *type* of action performed by the defendant. *Rose* (n 4) 541 (elsewhere) claimed that ‘*the fatal blow* … [must have been] necessary’; *Chisam* (n 17) 133 claimed that ‘There must be a reasonable necessity *for the killing*’; and *Faraj* (n 32) [8] (elsewhere) claimed that ‘the defendant [must] honestly believe that he needed to use *the threat of force*’ (emphases added).

62 Sangero (n 9) 144 describes the necessity requirement as involving a ‘comparison between [the defensive force that is exerted] and *the alternative ways of action available*’ (emphasis added), implying something like the specific action view. Sangero notices this problem, but claims that the necessity requirement is satisfied so long as D took a ‘reasonable way’ of acting. I critiqued that solution above.

63 Actions may be not equally justifiable for reasons other than the degree of force. For example, if D can repel C’s attack either by (i) touching C or (ii) sexually touching C (where these options involve equal force), then it would be unjustified to choose the sexual touching, for (*ceteris paribus*) that is more wrongful than battery.

64
*Weston* (n 3) 351; *Rose* (n 4) 541 (‘absolutely necessary’); *Palmer* (n 5) 832.

65
*Shaw* [2001] 1 WLR 1519 [19]. See also *Faraj* (n 32) [8]; *Rashford* (n 31) [19]; *Yaman* (n 31) [31]; *R (Collins)* (n 27) [60]; *Smith, Hogan, and Ormerod* (n 6) 401 and fn 340: ‘D must have a genuine *belief* in … the necessity of using force’, ‘D’s *belief* that the circumstances as he understands them render it … necessary for him to use force’; *Crown Court Compendium* (n 15) 18-1 [1], [16]–[17]: ‘Did D *believe* … that it was necessary to use force to defend themselves from an attack or imminent attack on them or others or to protect property or prevent crime? … [One condition of self-defence is D’s] *belief* that there is a need to use force … Self-defence does not apply if the jury are sure that D did not believe they needed to defend themselves’ (emphases added).

66
*Blackstone’s Criminal Practice* (n 6) A3.59: ‘were the facts (as D believed them to be) such that the use of force was necessary?’; *Simester and Sullivan* (n 6) 890: ‘The test … is partially subjective and partially objective: it asks whether, *on the facts as D (subjectively) honestly believed them to be*, the use of any force was *objectively* sufficiently necessary’ (Emphasis in the original).

67 Here, I assume that mistaken self-defence is not substantively distinct from non-mistaken self-defence. For an opposing view, see Andrew Simester, *Fundamentals of Criminal Law* (OUP 2021) ch 19. If the law (re)embraced the reasonable belief requirement, then this interpretation would become ‘(*reasonable* belief relative) factual necessity’.

68 Some commentators fail to notice this point when distinguishing factual from belief-relative formulations of the necessity requirement. JC Smith gives the example of a bodyguard shooting an assassin dead, where the bodyguard believed that deadly force was necessary, but a jury thought it was possible to take the assassin alive: JC Smith, ‘Using Force in Self-defence and the Prevention of Crime’ (1994) 47 CLP 101, 110. But the example requires elaboration. *On what basis* did the bodyguard believe that deadly force was necessary? If it was due to some underlying facts, eg that the assassin had such-and-such a weapon or would react in such-and-such a way, then we ask: if such facts were true, then would it have been necessary to use deadly force? If so, then *belief-relative factual necessity* would not rule out self-defence.

69 What if force was *factually* necessary, but D (incorrectly) lacked the belief that it was necessary? If *only* factual necessity was required, then D could use force in lawful self-defence without believing that any force was necessary, which is inappropriate. One response is to demand that D be aware of relevant justificatory circumstances if they are to gain the benefit of a defence: the *Dadson* principle (per *Dadson* (1850) 4 Cox CC 358), a view defended by John Gardner, *Offences and Defences: Selected Essays in the Philosophy of Criminal Law* (OUP 2007) ch 5.

70 In the language of the discussion in s 2A, I here assume that the necessity requirement is not being interpreted relative to *the end of rights vindication*, for one is at liberty to keep one’s phone out.

71 A belief about necessity is a belief about the (non-)availability of alternative options, which is a kind of fact. But it is not a ‘primary fact’ (about the surrounding circumstances). Rather, it is an evaluative ‘secondary fact’ (an inference drawn from the primary facts). Defendants may only rely on mistakes as to primary facts, not secondary facts. This is more intuitive, perhaps, regarding the standard of reasonableness. D may mistakenly believe her actions to be reasonable either because she is mistaken about the primary facts or because she is mistaken about what counts as reasonable. She may only rely on mistakes of the former kind. For discussion, see *Simester and Sullivan* (n 6) 792–3.

72 Defensive force is often described as having two ‘limbs’, viz a *subjective limb*: D is judged based on the facts as they subjectively believed them to be; and an *objective limb*: D must have acted objectively reasonably (relative to the facts as they subjectively believed them to be). Some locate the necessity requirement within the *subjective limb*: ‘It has become axiomatic that there are two basic questions to be answered in relation to self-defence … First, were the facts (as D believed them to be) such that *the use of force was necessary* (for the purpose claimed, e.g., for the purpose of self-defence) … Secondly, was the degree of force used reasonable for that purpose in the light of those perceived facts’: *Blackstone’s Criminal Practice* (n 6) A3.59 (emphasis added). Others locate the necessity requirement within the *objective limb*: ‘Force used in self-defence … must be “such force as is reasonable in the circumstances” if it is to be lawful … [Appraising whether force was reasonable] will involve questions concerning the *necessity* to use force … Force cannot be justified … to defend against an attack unless it was a sufficiently necessary means of doing so’: *Simester and Sullivan* (n 6) 890. It is tempting to think that those who locate the necessity requirement within the *subjective limb* must endorse *believed necessity* and that those who locate it within the *objective limb* must endorse *(belief-relative) factual necessity*. But this is not true. Framed within the *subjective limb*, the necessity requirement (under either formulation) becomes a freestanding condition of lawful self-defence. Framed within the *objective limb*, the necessity requirement (under either formulation) becomes a necessary condition of reasonableness, which, in turn, is a necessary condition of lawfulness. Unlike the choice between *believed necessity* and *belief-relative factual necessity*, the choice of whether to frame the necessity requirement within the *subjective* or *objective limb* makes no substantive difference.

73
*Simester and Sullivan* (n 6) 792–3. Famously, the law of dishonesty did formerly permit defendants to rely on mistaken beliefs about (social beliefs about) honesty in *Ghosh* [1982] QB 1053. The courts reverted to a more standard view, in which honesty was to be judged objectively (relative to the defendant’s perception of facts), in *Ivey v Genting Casinos* [2017] UKSC 67 and *Barton and Booth* [2020] EWCA Crim 575.

74 cf the position in German law, which does not traditionally contain a distinct proportionality requirement: Dubber and Hörnle (n 7) 407–8.

75 CJIA, s 76(7)(b) (emphasis added). I discuss the relevance of Parliament’s interventions in s 4.

76 CJIA, s 76(7)(a).

77 CJIA, s 76(7)(b).

78 In most similar cases, a defendant would not know for sure that force was unnecessary. The absence of ‘clean’ fact patterns like this might help to explain the absence of judicial scrutiny of the necessity requirement. It is unclear how many such cases exist. The absence of any direct authority on the necessity requirement may imply that such fact patterns are rare. But an alternative explanation is that the absence of critical attention to the necessity requirement has meant that lawyers and judges have simply failed to notice the salience of such fact patterns. That is the more plausible explanation regarding the reasonable belief requirement: once it became salient (after over a century of orthodox repetition), there was suddenly no shortage of cases involving unreasonable mistakes in self-defence.

79 I use ‘acceptable’ rather than ‘non-defeated’ to leave open whether ‘reasonable’ means ‘justified’. On this point, see the following two footnotes.

80 A point emphasised by John Gardner, ‘The Many Faces of the Reasonable Person’ (2015) 131 LQR 563. Gardner claims that the standard of reasonableness ‘sets no particular standard of justification’ (568); that it creates ‘a legally deregulated zone in which the many and varied underlying reasons are to be confronted by the decision-maker’ (572).

81 There are reasonableness factors besides necessity. For example, the fact that D *provoked* V is a factor militating against the reasonableness of D’s subsequent defensive force. But this fact does not automatically and conclusively determine that D’s defensive force was unreasonable. It is simply one factor among others. Defensive force can remain reasonable if V disproportionately responds to D’s provocation: *Keane, McGrath* (n 17).

82 Indeed, this assumes that ‘reasonable’ is synonymous with ‘justified on the balance of reasons’, per Gardner (n 80). But ‘reasonable’ is often used in a less demanding sense of being ‘close enough’ to justified (eg a ‘reasonable guess’ or ‘reasonable effort’). This is clearest in two-stage decision-making processes, where Person A decides a matter at first instance (eg an administrator), subject to review by Person B (eg a judge). Person B does not typically intervene merely because Person A made the wrong (unjustified) decision. Rather, Person B intervenes only where decisions are wrong *beyond a certain threshold*: not merely wrong, but *unreasonable*. For a sophisticated account of reasonableness in this sense, see Hasan Dindjer, ‘The Reasonable and the Justified’ in Ruth Chang and Amia Srinivasan (eds), *Conversations in Philosophy, Law, and Politics* (OUP 2024). While Dindjer focuses on two-agent cases, ‘reasonable’ in ordinary speech often takes this more permissive sense even in single-agent cases. For example, it may be unjustified (contrary to the balance of reasons) for me to eat cake at lunch (given my diet) yet still reasonable in the circumstances (given my stressful day).

83 CJIA, s 76(4)(b)(ii). The only exception is mistakes attributable to voluntary intoxication: s 76(5).

84 See n 8.

85 See eg David James Clark, ‘The Demands of Necessity’ (2023) 133 Ethics 473.

86 Quong (n 8) ch 5.

87
*Simester and Sullivan* (n 6) 890 (emphasis in original), citing *Williams (Demario)* [2020] EWCA Crim 193, [2020] Crim LR 637.

88
*Mutatis mutandis* for defence of another, defence of property, etc.

89 Irish law more clearly emphasises the importance of the purposive requirement. In *Commane* [1975] 1 Frewen 400, self-defence was unavailable to a defendant who first defended himself against the victim’s attack but subsequently strangled the unconscious victim, on the basis that the strangulation was not defensive at all. (Discussed in David Prendergast, ‘Defensive Killing by Initial Aggressors: *People (DPP) v Barnes* Revisited’ (2015) 54 IJ 115, 126.)

90 Compare the rebuttable presumptions of non-consent in s 75 of the Sexual Offences Act 2003 (as opposed to the *irrebuttable* presumptions in s 76 of that Act).

91
*Bayer* (n 45) [30]: ‘[was] the force … no more than was *reasonable* in all the circumstances[?]’; *Keane, McGrath* (n 17) [5]: ‘the … critical question for the jury is: was his response *reasonable* …?’ (emphases added). But note that *Keane* at [19] claims that ‘what is not unlawful is force which is reasonably necessary’. Many authorities include a *belief*-based necessity requirement (ie claim that D must believe that force was necessary (discussed in s 2C)) but formulate the *conduct*-evaluating test purely in terms of reasonableness, eg *Shaw* (n 65) [19]: ‘was the amount of force which he used reasonable?’. *Shaw* is noteworthy as it is a Privy Council case interpreting Belizean law, whose code justified the use of ‘*necessary* force or harm’ ([10]; emphasis added). Notwithstanding this, Belizean law was assumed to be the same as English law (per [14]) and best formulated in terms of reasonableness.

92 (Law Com No 218 (n 47) cl 27: ‘The use of force … if only such as is *reasonable* in the circumstances as [D] believes them to be, does not constitute an offence’ (emphasis added). Their omission of the necessity requirement was pointed out in Smith (n 68) 110.

93 Queensland’s Criminal Code Act 1899, s 271; Canadian Criminal Code 1985. s 34(1): ‘A person is not guilty of an offence if (a) they believe on reasonable grounds that force is being used against them or another person or that a threat of force is being made against them or another person.’ While this demands a reasonable belief, it does not contain a necessity requirement.

94 Rome Statute of the International Criminal Court, art 31(1)(c): ‘a person shall not be criminally responsible if … The person acts reasonably to defend himself … against an imminent and unlawful use of force in a manner proportionate to the degree of danger to the person’.

95 CJIA, s 76(1)(b) (emphasis added). This followed Parliament’s earlier approach to *public* defence in the CLA, s 3, viz: ‘A person may use such force as is *reasonable* in the circumstances in the prevention of crime … or in … lawful arrest’ (emphasis added). s 76 ‘applies where … the question arises’ as to whether D’s use of force was reasonable: CJIA, s 76(1). There is a heterodox argument that the common law test for private defence (eg self-defence) was not a test of reasonableness at all (but rather the twin tests of necessity and proportionality), and thus the question of whether D’s use of force was reasonable *simply does not ‘arise’*. But this would be an extreme interpretation of s 76. Parliament clearly presupposes that public and private defence share a common evaluative framework, a framework explicable in terms of reasonableness (a point assumed by Smith (n 68) 103: ‘the effect of these two sources of law [ie common law private defence versus statutory public defence] is at least broadly the same’). The question, then, is whether the reasonableness requirement *incorporates* the common law necessity (and proportionality) requirements, ie whether necessity (and proportionality) are necessary conditions of reasonableness.

96 CJIA, s 76(7)(b). This language is derived from the speech of Lord Morris in *Palmer* (n 5) 832.

97 Parliament did note a second relevant consideration, viz ‘that a person acting for a legitimate purpose may not be able to weigh to a nicety the exact measure of any necessary action’: CJIA, s 76(7)(a). But this seems to *presuppose* that D would be taking necessary action without *mandating* that this must be so. Similarly, the Explanatory Note to s 76 claims that ‘the defence will be available to a person if he honestly believed it was necessary to use force’. This might be read as Parliament presupposing the existence of a necessity requirement. But it does not say that self-defence is available *only* if D honestly believed in the necessity to use force, so does not strictly state a necessary condition of lawful self-defence. If anything, it implies a *sufficient* condition. But this cannot be right, for necessary *but grossly disproportionate* force is always unreasonable and unlawful. Given this unclarity, the Note should not be emphasised as an interpretive aid to the meaning of s 76.

98 CJIA, s 76(9). The exception to the merely clarificatory ambition is the ‘householder provisions’, which were explicitly intended to change the law.

99 CMV Clarkson, ‘Necessary Action: A New Defence’ [2004] Crim LR 81, 81–2.

100 ibid 81.

101
*Pace* Stephen’s famous formulation of a necessity defence in terms of doing ‘no more … than was *reasonably* necessary’ for the purpose of protecting against ‘inevitable and irreparable evil’, where the evil inflicted was ‘not disproportionate to the evil avoided’: James Fitzjames Stephen, *Digest of the Criminal Law* (4th edn, MacMillan and Co 1887) 24; this formulation was adopted by Brooke LJ in *Re A (children)* [2000] 4 All ER 961, [2001] Fam 147, 240.

102 On this view, a general necessity defence would concern strictly necessary responses to (even non-imminent) threats (judged relative to D’s reasonably believed facts, or perhaps simply the true facts), while self-defence would concern reasonable but not strictly necessary responses to imminent threats (judged relative to D’s honestly believed facts). In *Re A* (ibid), Brooke LJ thought self-defence was distinguished from necessity in requiring a response to (perceived) ‘unjust aggression’, but this requirement is doubtful given that the other judges thought (perhaps ‘quasi’) self-defence is available even against *innocent* threats (Ward LJ, 203–4; Walker LJ, 255), extended (albeit in obiter) to *non-threatening* bystanders in *Hichens* [2011] EWCA Crim 1626 [30].

